# Protocol to identify covalent inhibitors targeting RhoA Cys16

**DOI:** 10.1016/j.xpro.2026.104494

**Published:** 2026-04-14

**Authors:** Tin-Yan Koo, Chloe See-Hang Ho, Hillary Yui-Yan Yip, Clive Yik-Sham Chung

**Affiliations:** 1 School of Biomedical Sciences, Li Ka Shing Faculty of Medicine, The University of Hong Kong, Pokfulam Road, Hong Kong, China; 2 Department of Pathology, School of Clinical Medicine, Li Ka Shing Faculty of Medicine, The University of Hong Kong, Pokfulam Road, Hong Kong, China; 3 Centre for Oncology and Immunology, Hong Kong Science Park, Hong Kong, China

**Keywords:** Cell-based Assays, Cancer, Molecular/Chemical Probes, Proteomics

## Abstract

Ras homolog family member A (RhoA) is a therapeutic hotspot in many cancers but remains undruggable. Here, we present a protocol for identifying covalent inhibitors targeting RhoA Cys16. We describe steps for the identification of lead compounds by activity-based protein profiling (ABPP) screening and the validation of engagement by mass spectrometry and chemical biology experiments. We then detail procedures for investigating the effects of the compounds on RhoA signaling in cancer cells and mouse colorectal cancer (CRC) models.

For complete details on the use and execution of this protocol, please refer to Koo et al.^1^

## Highlights

• Detailed steps for identifying covalent inhibitors targeting RhoA Cys16
• Gel-based ABPP for identifying specific covalent ligands for RhoA over Rac1 and Cdc42
• Chemoproteomics and chemical biology experiments to examine target engagement in cancer cells
• Molecular and cellular assays to study RhoA-inhibitory and anticancer effects of the ligands

## Abstract (editor highlights)

Publisher’s note: Undertaking any experimental protocol requires adherence to local institutional guidelines for laboratory safety and ethics.

## Graphical abstract

**Figure:**
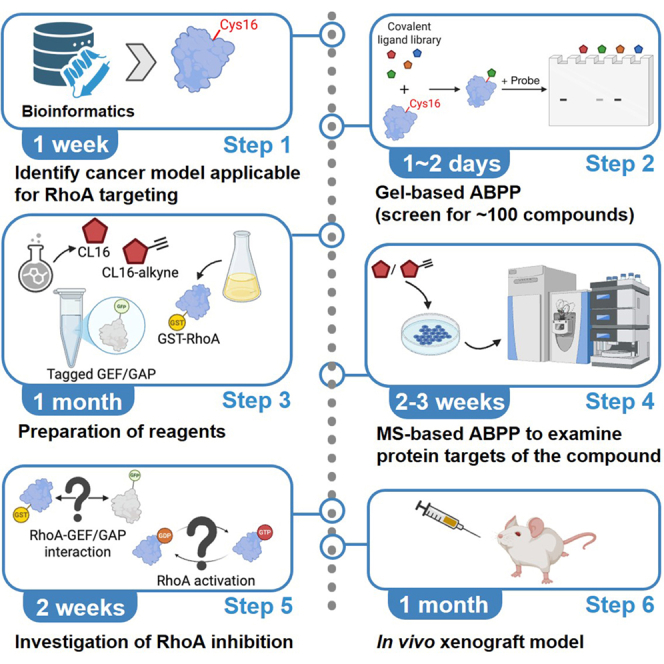

## Before you begin

Ras homolog family member A (RhoA) is a cellular small GTPase that governs proliferative signals and cytoskeletal dynamics. It is often upregulated in malignancies, such as breast, gastric and colorectal cancers (CRC), and hence is considered a therapeutic hotspot in oncology. However, due to its smooth surface and globular structure, RhoA has long been classified as an “undruggable” target. To address this challenge, covalent targeting of a unique cysteine on RhoA by covalent inhibitors, that generally exhibit stronger binding with the protein targets, could be an effective strategy. Recently, we identified that RhoA Cys16 is the unique cysteine in RhoA subfamily and is absent in other Rho GTPases. Notably, Cys16 is located adjacent to the functional domains (nucleotide-binding pocket and switch regions) of RhoA, suggesting the feasibility of modulating RhoA activity through small molecule binding to Cys16. We have developed a covalent inhibitor, CL16, targeting RhoA Cys16 in CRC cells. It significantly inhibits RhoA activity, leading to promising anti-cancer and anti-metastatic effects in CRC cells and mouse CRC models.

This protocol below describes the steps for identifying covalent inhibitors targeting RhoA Cys16 in CRC. It consists of: (1) Gel-based ABPP coupled with covalent ligand screening to identify lead compounds with specific binding to RhoA in vitro; (2) Mass spectrometry experiments to confirm RhoA Cys16 as the binding site of the lead compounds; (3) Molecular experiments to study the effects of the compounds on RhoA interactions with RhoGEF or RhoGAP expressed in CRC cells; (4) Cellular experiment to study the RhoA inhibitory effects of the compounds; (5) Mouse CRC xenograft models to assess antitumor effects of the lead compounds in vivo.

This protocol focuses on the identification of RhoA covalent inhibitors. Detecting and characterizing such inhibitors is crucial to decipher the activities and mechanisms of action of these synthetic compounds. These include probing the on- and off-target engagement of the inhibitors, as well as the biological effect. Similar strategies for identifying unique druggable sites and compound screening may be applicable to other proteins. Also, while the protocol describes specific steps to investigate RhoA activity in CRC cell lines, these experiments should be transferable to other adherent cell types.

### Innovation

It is the first protocol to provide step-by-step procedures for discovering RhoA covalent inhibitors targeting Cys16, one of the specific druggable hotspots on RhoA subfamily for cancer treatment. We describe a robust screening method, ABPP coupling with covalent ligand screening, to identify lead compounds with specific binding to RhoA in vitro. We also provide detailed procedures for multiple experiments to validate target engagement and examine the effects of the compounds in cancer cells and mouse models. This protocol complements a previously published protocol by Lin et al.^2^ While they described GTP exchange assay in detail, our protocol provides new information on the screening and target validation experiments by ABPP, chemoproteomics and chemical biology experiments. We also report other biochemical and cellular experiments which are important for evaluating the effects of the covalent inhibitors on RhoA activity and their anticancer properties.

### Institutional permissions (if applicable)

All animal experiments in this protocol were approved by the Committee of the Use of Live Animals in Teaching and Research (CULATR) at The University of Hong Kong and performed in accordance with the Animals (Control of Experiments) Ordinance of Hong Kong. Users of this protocol must confirm and obey corresponding institutional and governmental regulations.

### Identify a cancer model applicable to RhoA targeting

**Timing: 1 week**

We employed gene set enrichment analysis (GSEA) to identify a cancer model with significantly enriched RhoA-related pathway when comparing between early and advance tumour stages, and between tumours with high and low epithelial-mesenchymal transition (EMT) scores. Similar analysis may be performed for other cancer types by obtaining relevant data from The Cancer Genome Atlas (TCGA).

1. Extract data from TCGA database for colon adenocarcinoma (TCGA-COAD) and rectum adenocarcinoma (TCGA-READ).
  a. Build sample cohort with cancer type of interest from TCGA GDC Data Portal’s ‘cohort builder’ window.
    ***Note:*** When comparing between early (stage 0-I) and advance (stage IV) tumour stages, isolate the samples based on AJCC pathological stage by applying relevant filters under the ‘general diagnosis’ section.When comparing between high and low EMT scores, obtain all tumour samples and calculate EMT score as described in step 2.
  b. Download open-access RNA-Seq tumour data from TCGA under the ‘Repository’ widow by applying relevant filters under ‘access (open)’, ‘experiment strategy (RNA-seq)’, and ‘tissue type (tumour)’.
  c. Extract ‘tpm_unstrand’ matrix to obtain read counts normalised by TPM method.
2. Calculate EMT score via ‘hacksig’ package in R studio for all tumour samples.^3^
  a. Download and read EMT signature from https://www.gsea-msigdb.org/gsea/msigdb/human/geneset/HALLMARK_EPITHELIAL_MESENCHYMAL_TRANSITION.html. Set the list of genes as the signature.
  b. Assign each tumour samples an EMT score by using ssGSEA as the method when running the ‘hacksig’ package.^3^
  c. Organise samples based on EMT score. Separate into two groups based on the median EMT score.

***Note:*** Make sure the row name are gene symbols, and column names are sample names.

3. Run GSEA analysis.
  a. Download GSEA software from https://www.gsea-msigdb.org/gsea/index.jsp or utilise ‘GSEA’ package in R studio.^4^^,^^5^
  b. Run against ‘curated gene set’ in human collection.
  c. Investigate the normalised enrichment score (NES), nominal p-value, and FDR q-value for gene sets related to RhoA.

***Note:*** Examples of RhoA-related pathways: Biocarta Rho Pathway and Reactome RhoA.

***Note:*** Pathways with the highest upregulated NES, specifically with a nominal p-value and FDR q-value less than or equal to 0.05, are relevant enriched gene sets. Enrichment of RhoA-related pathway in tumour samples with advance pathological stage and/or tumour samples with high EMT score^6^^,^^7^^,^^8^ suggests the cancer type is a promising candidate for RhoA targeting.

### Preparation of recombinant proteins

**Timing: 3 days**

This section describes the steps for purifying GST-tagged protein, which is used for gel-based ABPP screening or co-immunoprecipitation.

4. Obtain pGEX-containing plasmids which are available from Addgene.

**Table:** 

Plasmids	Addgene #	Expressed proteins
pGEX-2T-RhoA-wt	12959	GST-RhoA
pGEX-2T-Cdc42-wt	12969	GST-Cdc42
pGEX-2T-Rac1-wt	12977	GST-Rac1

5. Express pGEX-containing plasmids in BL21(DE3) Escherichia coli expression system.
  a. Transform the recombinant plasmids into Escherichia coli expression strains BL21(DE3) and culture them in LB medium containing ampicillin (100 μg/mL) at 37°C for 16–18 h.
  b. Transfer 4 mL of the LB culture to 1 L of TB culture which contains ampicillin (100 μg/mL).
  c. Incubate the mixture at 37°C for 4–5 h until OD_600_ reaches ∼0.6.
    ***Note:*** Regularly monitor OD_600_ after 2.5 h to avoid overgrowth of the bacteria.
  d. Induce recombinant protein expression by adding 0.1 mM isopropyl β-D-1-thiogalactopyranoside (IPTG) and incubate the mixture at 18°C for 16–18 h.
    ***Note:*** The typical working concentration of IPTG to induce recombinant GST-tag protein expression ranges from 0.1 to 1 mM, and the induction time is 12–16 h at 18°C. Optimization may be required for different plasmids.
6. Purify recombinant proteins using affinity GST-tag.
  a. Harvest bacterial pellet by centrifugation at 4,000 × *g* at 4°C for 20 min.
  b. Add 5 mL of B-PER™ Pierce™ Complete Bacterial Protein Extraction Reagent supplemented with protease inhibitor cocktail per gram of bacterial pellet.
    ***Note:*** Follow individual manufacturer’s instruction. Pierce Protease and Phosphatase Inhibitor Mini Tablets are used in this protocol at the concentration of 1 tablet/10 mL. User may also use their own in-lab recipe.
    **CRITICAL:** Ensure complete resuspension of bacterial pellet by vigorous vortexing.
  c. Clarify the bacterial lysate by centrifugation at 16,000 × *g* at 4°C for 30 min. Save the supernatant.
  d. Incubate bacterial lysate with pre-equilibrated glutathione-agarose beads for 1 h at 4°C with gentle end-to-end rotation.
    i. Prepare beads by adding 10× bed-volume of washing buffer, and pellet beads by centrifugation at 700 × *g* at 4°C for 3 min.
    ii. Add 10× bed-volume of supernatant to the beads.
      ***Note:*** This protocol uses a bead: bacterial lysate ratio of 1:10. Other ratios may be used for other constructs with different expression efficiency.
  e. Wash beads 5–7 times with washing buffer to remove nonspecific proteins.
    i. Pellet beads by centrifugation at 700 × *g* at 4°C for 3 min and discard supernatant. Apply new 10× bed-volume of washing buffer. Repeat the washing step.
    ii. Monitor the protein concentration of supernatant using nanodrop.
    iii. A zero or negative Abs280 reading indicates sufficient washing.
7. Elute and characterize recombinant proteins.
  a. Apply freshly prepared elution buffer to the beads.
  b. Pellet beads by centrifugation at 3,000 × *g* at 4°C for 3 min. Collect eluant.
  c. Repeat if necessary.
  d. Measure the protein concentration using nanodrop.

***Note:*** Check Abs260/280 ratio to detect whether there is any nucleic acid contamination. A pure protein sample should have a value of ∼0.6.

8. Clean up proteins by dialysis to remove excess glutathione molecules.
  a. Pre-wet dialysis cassette (7K MWCO) with PBS or exchange buffer.
  b. Load protein samples into the cassette.
  c. Incubate the cassette in PBS with stirring at 4°C for 1 h. Replace the solution with new PBS every hour and then incubate for 16–18 h.
  d. Collect the protein in the cassette using a syringe.
  e. Quantify the protein amount (e.g., by Bradford, bicinchoninic acid assay (BCA) or Lowry assay).
9. Determine protein purity by protein staining after gel electrophoresis.
  a. Aliquot 30 μg protein samples and denature samples by boiling in reducing sampling buffer for 5 min.
  b. Resolve the protein by gel electrophoresis.
  c. Visualize protein bands by Coomassie blue or silver staining depending on amount of protein loading. The three purified proteins are expected to show a single band at ∼47 kDa.

### Preparation of cell lysates with RhoGEF or RhoGAP overexpression

**Timing: 4 days**

These steps outline the procedures to overexpress guanine nucleotide exchange factors (GEFs) or GTPase activating proteins (GAPs) in HEK293T cells for subsequent co-immunoprecipitation experiments.

10. Obtain the following plasmids from Addgene.

**Table:** 

Plasmids	Addgene #
pEGFP-FLAG-AKAP-Lbc	67571
pDEST-EGFP_Hu_p115-RhoGEF (WT)	136337
pCAG-mCherry-FKBP-GAP	222632
pEGFP-SRGAP1	187269

***Note:*** This protocol uses these few constructs as an illustration. Users may perform cloning to generate tagged GEFs or GAPs plasmids to investigate interactions of RhoA with other proteins which are not commercially available.

11. Seed HEK293T cells into a 10-cm culture dish and incubate for 16–18 h at 37°C under 5% CO_2_.
12. Transfect one 10-cm dish of HEK293T cells at 50%–60% confluency with a DNA-lipid mixture.
  a. Prepare Tube A (60 μg of DNA plasmid in 500 μL of Opti-MEM) and Tube B (60 μL of lipofectamine 2000 in 500 μL of Opti-MEM).
  b. Incubate each tube for 10 min at 20°C–25°C.
  c. Gently add content of Tube B into Tube A. Mix by slow pipetting or inversion of the tubes.
    **CRITICAL:** Do not vortex the DNA-lipid mixture. In this mixing step, the lipid and nucleic acid form cationic complexes for cargo delivery. Excessive agitation can disrupt these complexes, leading to a reduced transfection efficiency and potential toxicity.
  d. Incubate the solution mixture for 20 min at 20°C–25°C.
  e. Add the DNA-lipid mixture to HEK293T cells dropwise and incubate the cells at 37°C in a 5% CO_2_ atmosphere for 48 h.
    **CRITICAL:** Observe cell density under a microscope. Cells should reach *ca.* 50%–60% confluency on the day of transfection for optimal transfection efficiency.
    ***Note:*** 48–72 h of post-transfection is generally optimal for harvesting cells. Protein expression levels begin to decline beyond this point. Assess transfection efficiency by observing GFP or RFP signal under a microscope. The optimal time for protein expression may vary for different constructs.
13. Harvest RhoGEF- or RhoGAP-overexpressing cell lysates in exchange buffer.
  a. Aspirate cell culture medium.
  b. Wash cells twice with pre-chilled PBS.
  c. Harvest cells by scrapping in exchange buffer.
  d. Probe-sonicate the cell suspension for 15 s at 15% power.
  e. Clarify lysate by centrifugation at 13,200 × *g* for 5 min. Save the supernatant.
  f. Quantify protein concentration (e.g., Bradford, BCA, Lowry assay) and normalize protein samples.

***Note:*** Prior testing may be required to ensure complete protein extraction from cell suspension by probe sonication, as efficiency can vary among different sonicator models.

### Preparation of CL16 and CL16-alkyne

**Timing: 2**–**3 days for CL16 and 4**–**5 days for CL16-alkyne**

**Figure 1:**
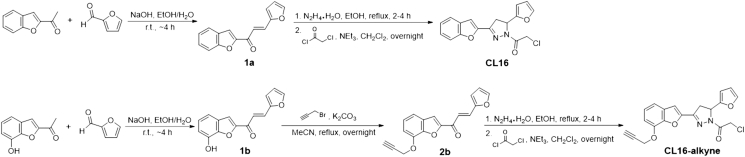
Synthetic scheme of CL16 and CL16-alkyne

This section outlines the synthesis of CL16, and CL16-alkyne used in LC-MS/MS experiment (Figure 1). Details and characterization data are provided in the supplementary data by Koo et al.^1^ For other cysteine-reactive covalent ligands, they are available from various vendors. We used the compounds from Enamine in our reported screening experiments.^1^

14. Synthesize compound 1a according to reported literature.^9^ Add furfural (1 equiv.) and 2-acetylbenzofuran (1 equiv.) to sodium hydroxide (1.25 equiv.) in ethanol/water (1:1). Allow reaction to complete, extract and purify the organic product.
15. Add hydrazine (2.0 equiv.) to 1a in ethanol and heat under reflux. Extract the organic intermediate and proceed without further purification.

***Note:*** Hydrazine and its derivatives are highly toxic, corrosive, strongly reducing and potentially carcinogenic. Handle reagents in a certified fume hood with adequate ventilation.

16. Add chloroacetyl chloride (1.2 equiv.) and triethylamine (2.0 equiv.) in dichloromethane to allow reaction on ice for 16 h. Extract and purify the organic product to yield CL16.

***Note:*** Chloroacetyl chloride is highly toxic and corrosive. Handle reagents in a certified fume hood with adequate ventilation.

17. Synthesize compound 1b according to step 1 except using 2-acetyl-7-hydroxybenzofuran instead of 2-acetylbenzofuran.
18. Add propargyl bromide (1.5 equiv.) and potassium carbonate (2.5 equiv.) to compound 1b (1.0 equiv.) in acetonitrile. Extract and purify the organic product to yield compound 2b.
19. Synthesize CL16-alkyne according to steps 2 and 3 except using compound 2b instead of compound 1a.

## Key resources table

**Table:** 

REAGENT or RESOURCE	SOURCE	IDENTIFIER
**Antibodies**		

RhoA (67B9) Rabbit mAb (Dilution 1:1000 for WB)	Cell Signaling Technology	Cat# 2117S; RRID:AB_10693922
Anti-GFP (D5.1) XP® (Dilution 1:1000 for WB)	Cell Signaling Technology	Cat# 2956S; RRID:AB_1196615
Rac1/2/3 Antibody (Dilution 1:1000 for WB)	Cell Signaling Technology	Cat# 2465S; RRID:AB_2176152
Cdc42 Antibody (Dilution 1:1000 for WB)	Cell Signaling Technology	Cat# 2462S; RRID:AB_2078085
Anti-rabbit IgG HRP (Dilution 1:3000 for WB)	Cell Signaling Technology	Cat# 7074S; RRID:AB_2099233

**Bacterial and virus strains**		

BL21(DE3) Competent Cells	Thermo Scientific™	Cat# EC0114

**Chemicals, peptides, and recombinant proteins**		

20× LumiGLO® Reagent and 20× Peroxide	Cell Signaling Technology	Cat# 7003P
Dithiothreitol (DTT)	Aladdin	Cat# D265376
Bromophenol blue	Macklin	Cat# B802654
DMEM, powder, high glucose	Gibco	Cat# 12100046
RPMI 1640 Medium, powder, HEPES	Gibco	Cat# 23400021
Fetal Bovine Serum (FBS)	Gibco	Cat# A5256701
Penicillin-Streptomycin (10,000 U/mL)	Gibco	Cat# 15140122
Gibco trypan Blue Solution, 0.4%	Thermo Scientific™	Cat# 15250061
Pbs (10×), pH 7.4	Gibco	Cat# 70011044
Trypsin-EDTA (0.25%), phenol red	Gibco	Cat# 25200072
Sodium bicarbonate (NaHCO_3_)	Macklin	Cat# S818080
Sodium chloride (NaCl)	Macklin	Cat# S799334
Ultrapure™ Tris	Thermo Scientific™	Cat# 15504020
Reduced glutathione	Macklin	Cat# R917465
HEPES pH 7.4	Macklin	Cat# M718430
Magnesium chloride (MgCl_2_)	Beyotime	Cat# R0058
Urea	Aladdin	Cat# U111903
Sodium Dodecyl Sulfate (SDS)	Thermo Scientific™	Cat# 28312
Acetone	VWR	Cat# VWR-20066.330
Acetonitrile (MeCN)	Macklin	Cat# A742132
Dimethyl Sulfoxide (DMSO)	Adamas	Cat# 01375909
Lipofectamine 2000 Transfection Reagent	Thermo Scientific™	Cat# 11668030
Opti-MEM™ I Reduced Serum Medium	Thermo Scientific™	Cat# 31985088
Ampicillin	Sigma-Aldrich	Cat# A5354
Isopropyl β-D-1-thiogalactopyranoside (IPTG)	Sigma-Aldrich	Cat# I6758
BD Difco Terrific Broth	Thermo Scientific™	Cat# DF0438-17
Luria Broth Base (Miller’s LB Broth Base)	Invitrogen™	Cat# 12795027
Glycerol	Macklin	Cat# G810580
B-PER™ Pierce™ Complete Bacterial Protein Extraction Reagent	Thermo Scientific™	Cat# 89821
Pierce Protease and Phosphatase Inhibitor Mini Tablets, EDTA-free	Life Technologies	Cat# A32961
Streptavidin-Agarose beads	Thermo Scientific™	Cat# 20353
Glutathione-Agarose beads	Thermo Scientific™	Cat# 16100
Rhosin	Sigma-Aldrich	Cat# 555460
Iodoacetamide	Sigma-Aldrich	Cat# I6125
Copper(II) sulfate pentahydrate	Sigma-Aldrich	Cat# 678937
Tris(2-carboxyethyl)phosphine hydrochloride (TCEP)	Sigma-Aldrich	Cat# C4706
Tris[(1-benzyl-1H-1,2,3-triazol-4-yl)methyl]amine (TBTA)	Cayman Chemical	Cat# 18816
Desthiobiotin-PEG-Azide (DTB-PEG-N_3_)	Sigma-Aldrich	Cat# 902020
Iodoacetamide-rhodamine (IA-Rh)	Setareh Biotech	Cat# 6222
PEG400	Sigma-Aldrich	Cat# 202398
Tween80	Thermo Scientific™	Cat# 28329
Corning® Matrigel® Basement Membrane Matrix, ∗LDEV-Free	Corning	Cat# 356234
Methanol	Macklin	Cat# M813903
Furfural	Macklin	Cat# F809411
2-acetylbenzofuran	Macklin	Cat# A687435
Sodium hydroxide AR	Dieckmann	Cat# MC10003
Ethanol	Sigma-Aldrich	Cat# 459844
Hydrazine	Aladdin	Cat# H432580
Chloroacetyl chloride	Dieckmann	Cat# MC20923
Dichloromethane	Duksan	Cat# 2965
Triethylamine	Macklin	Cat# T818772
2-acetyl-7-hydroxybenzofuran	Aladdin	Cat# A164473
Propargyl bromide	Macklin	Cat# P815876
Potassium carbonate	Aladdin	Cat# P432991

**Critical commercial assays**		

RhoA Pull-down Activation Assay Biochem Kit (bead pull-down format)	Cytoskeleton	Cat# BK036
Rac1 Pull-Down Activation Assay Biochem Kit	Cytoskeleton	Cat# BK035
Pierce™ BCA Protein Assay Kit	Thermo Scientific™	Cat# 23225
Pierce™ Silver Stain Kit	Thermo Scientific™	Cat# 24612

**Experimental models: Cell lines**		

Human: HEK293T (Wildtype/Fetus/Female)	ATCC	#CRL-3216
Human: HCT116 (Wildtype/48Y/Male)	ATCC	#CCL-247
Human: HT29 (Wildtype/44Y/Female)	ATCC	#HTB-38
Human: SW620 (Wildtype/51Y/Male)	ATCC	#CCL-227

**Experimental models: Organisms/strains**		

Mouse: BALB/c nude mice; 6‒8-weeks-old, male	Centre for Comparative Medicine Research, HKU	RRID: MGI:2161072

**Oligonucleotides**		

pGEX-2T-RhoA-wt	A gift from Gary Bokoch	Addgene# 12959
pGEX-2T-Cdc42-wt	A gift from Gary Bokoch	Addgene# 12969
pGEX-2T-Rac1-wt	A gift from Gary Bokoch	Addgene# 12977
pEGFP-FLAG-AKAP-Lbc	Burmeister et al.^10^	Addgene# 67571
pDEST-EGFP_Hu_p115-RhoGEF (WT)	de Carcer et al.^11^	Addgene# 136337
pCAG-mCherry-FKBP-GAP(ARHGAP29)	Marshall-Burghardt et al.^12^	Addgene# 222632
pEGFP-SRGAP1	Lian et al.^13^	Addgene# 187269

**Deposited data**		

Proteomic data	Koo et al.^1^	PRIDE ID: PXD053036 and PXD053041

**Software and algorithms**		

MaxQuant v2.0.3.0	Max-Planck-Institute of Biochemistry	https://www.maxquant.org/
MSFragger v3.7	Nesvizhskii lab	https://msfragger.nesvilab.org/
ImageJ	Available from NIH	https://imagej.net/software/fiji/
RStudio version 1.4.1106	RStudio	https://www.rstudio.com/

**Other**		

Sequencing Grade Modified Trypsin	Promega	Cat# V5111
C18 Stage Tips	Thermo Scientific™	Cat# 87782
Micro Bio-Spin Chromatography Columns	Bio-Rad	Cat# 7326204
Aurora C18 UHPLC column (75 μm i.d. × 25 cm length × 1.6 μm particle size)	IonOpticks Australia	https://ionopticks.com/product/aurora-ultimate-25cm-x-75um-c18-uhplc-column/

## Materials and equipment

### LB broth

Dissolve 25 g of LB powder in 1 L of MilliQ water and autoclave the media at 121°C for 30 min. It can be stored for up to 1 year at 25°C. Add ampicillin dissolved in MilliQ water (final concentration: 100 μg/mL) before use.

### TB broth

Dissolve 47.6 g of TB powder and 4 mL of glycerol in every 1 L MilliQ water and autoclave the media at 121°C for 30 min. It can be stored for up to 1 year at 25°C. Add ampicillin dissolved in MilliQ water (final concentration: 100 μg/mL) before use.

### Culture medium

Dissolve the medium base powder and NaHCO_3_ in MilliQ water. Filter the medium with a sterile 0.2 μm membrane before addition of FBS and Penicillin-Streptomycin. Prepare 1 L and it can be stored for up to a month at 4°C.

**Table:** 

Reagent	Final concentration	Amount
NaHCO_3_	RPMI: 2.0 g/L or DMEM: 3.7g/L	Various
Fetal bovine serum	10 vol%	100 mL
Penicillin-Streptomycin (10,000 U/mL)	1 vol%	10 mL
MilliQ water	–	890 mL
**Total**	–	**1 L**

***Note:*** Use RPMI medium for HCT116 or SW620 cells and DMEM for HEK293T or HT29 cells. For other cell lines, check the recommended medium and adjust the amount of NaHCO_3_ according to manufacturer’s instruction.

### 4× reducing sampling buffer

Add the content accordingly and place on shaker for 16–18 h for even mixing. It can be stored for up to 1 year at 4°C.

**Table:** 

Reagent	Final concentration	Amount
Tris-HCl pH 6.5	0.2M	10 mL
Dithiothreitol (DTT)	0.4M	20 mL
Sodium dodecyl sulfate (SDS)	8%	4 g
Bromophenol blue	0.4%	200 mg
Glycerol	32%	16 mL
MilliQ water	N/A	4 mL
**Total**	–	**50 mL**

### Washing buffer

Dissolve the salt and adjust pH accordingly. Prepare 1 L and it can be stored for up to 1 year at 25°C.

**Table:** 

Reagent	Final concentration	Amount
NaCl	150 mM	8.770 g
Tris pH 8.0	50 mM	6.057 g
MilliQ water	N/A	1L
**Total**	–	**1 L**

### Elution buffer

Freshly dissolve reduced glutathione in washing buffer before use.

**Table:** 

Reagent	Final concentration	Amount
Reduced glutathione	10 mM	61.464 mg
Washing buffer	N/A	20 mL
**Total**	–	**20 mL**

### GTP-exchange buffer

Dissolve the salt and adjust pH accordingly. Prepare 1 L and it can be stored for up to 1 year at 25°C.

**Table:** 

Reagent	Final concentration	Amount
HEPES pH 7.4	20 mM	95.32 mg
NaCl	150 mM	175.32 mg
MgCl_2_ (aq)	5 mM	9.521 mL
MilliQ water	N/A	10.479 mL
**Total**	–	**20 mL**

### TCEP solution

Freshly dissolve TCEP in MilliQ water before use.

**Table:** 

Reagent	Final concentration	Amount
TCEP	110 mM	31.5 mg
MilliQ water	N/A	1 mL
**Total**	–	**1 mL**

### IA solution

Freshly dissolve IA in MilliQ water before use.

**Table:** 

Reagent	Final concentration	Amount
IA	100 mM	18.5 mg
MilliQ water	N/A	1mL
**Total**	–	**1 mL**

### Urea denaturing solution

Freshly dissolve urea in PBS. Dilute 3-fold using PBS to obtain a 2M urea solution.

**Table:** 

Reagent	Final concentration	Amount
Urea	6M	3.6 g
PBS	N/A	10 mL
**Total**	–	**10 mL**

### SDS denaturing solution

Dissolve SDS powder in PBS. It can be stored for up to 1 year at 25°C. A 0.2% (w/v) SDS solution can be prepared by diluting with PBS.

**Table:** 

Reagent	Final concentration	Amount
SDS	1.2% (w/v)	1.2 g
PBS	N/A	100 mL
**Total**	–	**100 mL**

### Click master mix solution

Prepare stocks of copper (II) sulfate pentahydrate (CuSO_4_5H_2_O), tris[1-benzyl-1*H*-1,2,3-triazol-4-yl) methyl]amine (TBTA) and desthiobiotin-PEG_3_-azide (DTB-PEG-N_3_). They can be stored for up to 1 year at −20°C. Freshly prepare TCEP solution by dissolving TCEP powder in MilliQ water.

**Table:** 

Reagent	Final concentration	Amount for (*n*=1)
CuSO_4_· 5H_2_O (50 mM in MilliQ water)	1 mM	40 μL
TBTA (1.7 mM in DMSO/*tert*-butanol (1:4, v/v))	100 μM	120 μL
DTB-PEG-N_3_ (5 mM in DMSO)	100 μM	40 μL
TCEP (50 mM in MilliQ water)	1 mM	40 μL
**Total**	–	**240 μL**

***Note:*** Prepare enough master mix solution for at least *n* + 2 samples.

**CRITICAL:** Follow the above addition sequence for each reagent. Vortex briefly to mix well at each step.

### LC-MS/MS buffer A

Prepare Buffer A accordingly. Prepare enough volume for each experiment.

**Table:** 

Reagent	Final concentration	Amount
Water	98% (v/v)	979 mL
Acetonitrile	2% (v/v)	20 mL
Formic acid	0.1% (v/v)	1 mL
**Total**	–	**1 L**

### LC-MS/MS buffer B

Prepare Buffer B accordingly. Prepare enough volume for each experiment.

**Table:** 

Reagent	Final concentration	Amount
Acetonitrile	99.9% (v/v)	999 mL
Formic acid	0.1% (v/v)	1 mL
**Total**	–	**1 L**

### PET solution

Add the content accordingly and place on shaker for 16–18 h for even mixing. It can be stored for up to 1 year at 25°C.

**Table:** 

Reagent	Final concentration	Amount
PEG400	60% (v/v)	30 mL
Ethanol	30% (v/v)	15 mL
Tween80	10% (v/v)	5 mL
**Total**	–	**50 mL**

**Table:** 

EquipmentModel	Brand	Product website
timsTOF Pro	Bruker	https://www.bruker.com/en.html
Savant SPD131DDA-115 SpeedVac Concentrator	Thermo Scientific™	https://www.thermofisher.com/hk/en/home.html
Perkin Elmer	Victor 3	https://www.perkinelmer.com/category/analysis-characterization
ChemiDoc Touch Imaging System	Biorad	https://www.bio-rad.com/en-hk/product/chemidoc-touch-imaging-system?ID=NINJCT4VY
NanoDrop™ 2000/2000c Spectrophotometers	Thermo Scientific™	https://www.thermofisher.com/order/catalog/product/ND-2000

## Step-by-step method details

### Gel-based activity-based protein profiling (ABPP) for screening

**Timing: ∼10 h for the 2-round screening of ∼100 compounds to identify lead compounds binding to RhoA**

**Figure 2:**
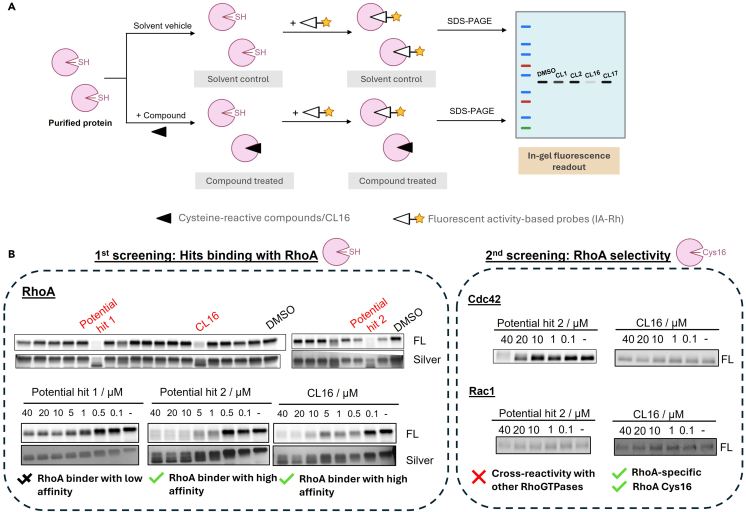
Identification of CL16 binding to RhoA by gel-based ABPP experiment (A) Rationale of gel-based ABPP for screening compound binding to RhoA *in vitro*. Purified RhoA protein was pre-treated with solvent control or library compounds followed by labelling with fluorescent activity-based probes. (B) Expected outcomes in gel-based ABPP. In the 1^st^ round of screening, compound binding to RhoA results in a decrease in in-gel fluorescence (FL) intensity. Further validation of the binding can be performed by experiments using varying concentrations of the hit compounds. In the 2^nd^ round of screening, selected hits are examined for their binding to Cdc42 and Rac1 to determine their selectivity for RhoA.^1^

In these steps, we describe the use of ABPP to identify covalent ligands targeting RhoA Cys16 *in vitro*. RhoA Cys16 is a unique druggable cysteine on RhoA subfamily, as revealed by sequence alignment analysis with other Rho GTPases. The two-round workflow enables the identification of lead molecules targeting RhoA, specifically at Cys16 (Figure 2). The same rationale can be extended to other protein targets.

1. Thaw GST-RhoA protein on ice and dilute it to 4 ng/μL using 1× PBS.
2. Aliquot 25 μL of protein per sample (i.e., 0.1 μg protein).

**CRITICAL:** This amount of protein is enough to be visualized on gel.

3. Add 1 μL of cysteine-reactive compounds (4 mM in DMSO; final concentration: 20 μM) or DMSO as control to each protein sample.
4. Incubate at 20°C–25°C for 1 h with constant agitation.

***Note:*** If shaker is not available, vortex every 15 min to ensure thorough mixing of the protein and the compound.

5. Add 1 μL of iodoacetamide-rhodamine (IA-Rh) (25 μM in DMSO; final concentration: 1 μM) to each sample. Incubate at 20°C–25°C in dark with constant agitation.

***Note:*** IA-Rh is light-sensitive, stored in dark or wrapped with aluminum foil.

6. Boil samples in 4× reducing sampling buffer at 90°C for 5 min.
7. Resolve proteins bands by gel electrophoresis.
8. Record in-gel fluorescence intensity using ChemiDoc Touch Imaging System (Rhodamine channel).

***Note:*** Rinse the gel thoroughly with distilled water to wash away residual IA-Rh on the gel surface, which may result in high background noise.

9. Silver stain the gel for visualization of protein content.
10. Identify potential hit compounds for further testing.
  a. Measure in-gel fluorescence intensity (FL) and the band intensity from silver staining (Silver) using ImageJ.
  b. Determine the intensity ratio of FL/Silver.
  c. Compounds showing 30% or less intensity ratio compared to control samples are considered as lead compounds.

***Note:*** This protocol uses 30% as the cut-off value. This number can vary depending on the testing molecules and protein of interest.

11. Perform the 2^nd^ screening experiment.
  a. Run the gel-based ABPP experiments of the lead compounds identified in step 10 using GST-RhoA, GST-Rac1 and GST-Cdc42 proteins, respectively.
  b. Use a concentration gradient of test compounds (final concentration: 0 – 20 μM) to examine compound binding to these proteins at different concentrations.
12. Compare IC_50_ values of the compounds with the 3 Rho GTPases.
  a. Determine the intensity ratio of FL/Silver as described in Step 10a and 10b.
  b. Plot the intensity ratio of FL/Silver against compound concentrations in GraphPad Prism.
  c. Fit the value to one-phase decay curve and obtain the IC_50_ value.

***Note:*** Compounds showing low IC_50_ for GST-RhoA and much higher IC_50_ values for GST-Rac1 and GST-Cdc42 are considered selective binders to RhoA *in vitro*.

### LC-MS/MS detection of compound binding to RhoA Cys16

**Timing: Sample preparation, 3 days; detection and analysis, variable**

**Figure 3:**
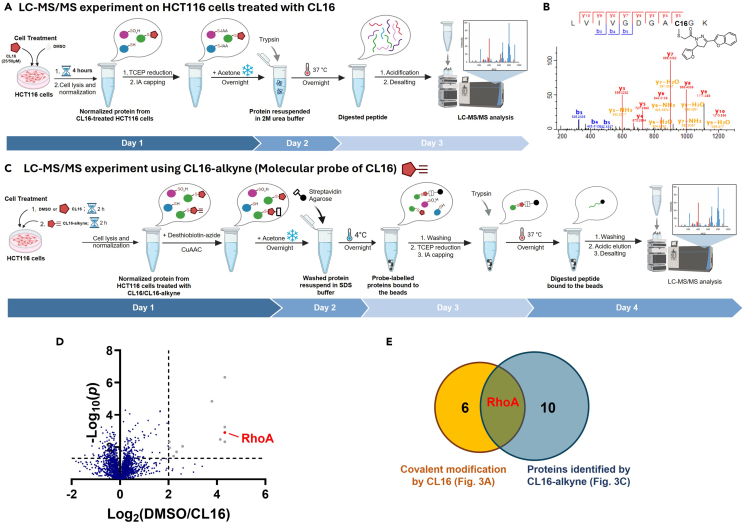
Validation of CL16-RhoA binding by LC-MS/MS experiments (A) Workflow of LC-MS/MS experiments to detect RhoA-CL16 engagement in HCT116 cells. CL16-treated cells were harvested, digested and analyzed by LC-MS/MS. (B) Representative MS/MS showing CL16 modification on RhoA Cys16 in HCT116 cells.^1^ (C) Workflow of LC-MS/MS experiments using CL16-alkyne (a CL16-molecular probe) to study target profile of CL16. (D) Volcano plot revealing protein targets of CL16 identified by CL16-molecular probe.^1^ Statistical analyses were performed by two-tailed Student’s t-test by MS Excel. (E) Venn diagram summarizing the protein targets of CL16 identified in (A) and (C), highlighting RhoA as the primary target.

This section outlines the procedure to detect binding of covalent ligand to RhoA Cys16 in HCT116 cells by LC-MS/MS. CL16, identified from gel-based ABPP, is used here as the example for illustration. The aim of MS experiment herein is to validate the engagement between RhoA and CL16 in CRC cells. Briefly, proteins are harvested from CL16-treated cells and prepared for MS on Day 1 (Step 13–17). On Day 2 (Step 18–20), the proteins are resuspended and digested into peptides. On Day 3 (Step 21–25), digested peptides are collected for LC-MS/MS analysis, followed by analysis in Step 26–27. Figure 3A provides a schematic representation of this experiment. Sample preparation in other models or using different compounds may require optimization.

#### Day 1

13. Incubate HCT116 cells with complete culture medium supplemented with DMSO or CL16 (25 or 50 μM) for 4 h at 37°C under 5% CO_2_.
14. Prepare cell lysates in PBS (2 mg/mL; 100 μL per sample).
  a. Aspirate medium.
  b. Wash cells twice with pre-chilled PBS.
  c. Harvest cells in PBS by scrapping.
  d. Probe-sonicate the cell suspension for 15 s at 15% power.
  e. Clarify the lysates by centrifugation at 13,200 × *g* for 5 min. Save the supernatant.
  f. Quantify protein concentration (e.g., by Bradford, BCA or Lowry assay) and normalize protein sample.

***Note:*** Prior testing may be required to ensure complete protein extraction from cell suspension by probe sonication due to different models of sonicators being used.

15. Add 50 μL of freshly prepared TCEP solution to the cell lysates. Incubate at 65°C for 30 min.
16. Add 50 μL of freshly prepared IA solution to the mixture. Incubate at 37°C for 30 min in the dark.
17. Precipitate proteins with 600 μL pre-chilled acetone at −20°C for 16–18 h.

***Note:*** This is an optional pause point. However, long-term storage of protein in acetone may cause denaturation. This protocol has not tested protein kept in acetone for longer than a weekend.

#### Day 2

18. Centrifuge samples at 13,200 × *g* for 10 min to precipitate proteins. Discard the supernatant.
19. Resuspend protein pellet in 100 μL of freshly prepared 2M urea denaturing solution.
20. Digest samples with 4 μL trypsin solution at 37°C for 16–18 h with constant agitation.
  a. Reconstitute 20 μg lyophilized sequencing grade modified trypsin powder with 40 μL reconstitution buffer.
  b. Incubate on ice for 5 min.
  c. Pipette up and down to ensure good mixing before use.

***Note:*** Optimal protein digestion time is between 16 and 21 h. Prolonged incubation may cause over-digestion, while insufficient incubation may result in incomplete peptide digestion and reduce peptide detection.

#### Day 3

21. Acidify samples with 5 μL formic acid (final concentration: 5%).
22. Centrifuge samples at 13,200 × *g* for 30 min to remove any precipitate. Collect the supernatant.
23. Dry the peptide solution with vacuum concentrator.
24. Desalt the peptides with C18 Stage tips *as per* manufacturer’s instruction.
25. Analyze peptide on Aurora C18 UHPLC column (75 μm i.d. × 25 cm length × 1.6 μm particle size (IonOpticks Australia) coupled to timsTOF Pro mass spectrometer (Bruker).
  a. Chromatographic separation can be achieved by the following gradients. The composition of Buffer A and Buffer B changes linearly over time.

**Table:** 

Time (min)	Duration (min)	Flow rate (nL/min)	Buffer A/%	Buffer B/%
0	-	300	98	2
90	90		70	30
115	25		50	50
123.5	8.5		5	95
124	0.5		98	2
129.5	5.5		98	2
  b. Collect MS data over a m/z range of 100 to 1700, and MS/MS range of 100 to 1700 with 1.1s for each TIMS cycle. Include 1 MS + an average of 10 PASEF MS/MS scans.
26. Search the MS raw files using MaxQuant (v2.0.3.0) with Uniprot human database (UP000005640).
  a. Specify digestion with trypsin with up to 3 missed cleavages.
  b. Include cysteine carbamidomethylation (+57.02146) as fixed modification, N-terminal acetylation (+42.01057), methionine oxidation (+15.99491) and CL16-cysteine adduct (+235.06333) as variable modifications.
  c. Set peptide false discovery rate to 1%.

***Note:*** Latest version of software or another search engine can be used.

***Note:*** Mass shift for cysteine modifications by other ligands needs to be calculated in accordance with molecules’ structures.

27. Identify CL16-modified peptides.
  a. Filter out peptides with spectral count less than 2, PEP larger than 0.01 or a lower spectral count at 50 μM CL16 treatment compared to 25 μM.
  b. Visualize the MS/MS. A representative example showing CL16 modification at RhoA Cys16 is provided in Figure 3B.

### LC-MS/MS detection of RhoA-CL16 engagement with CL16-alkyne

**Timing: Sample preparation, 4 days; detection and analysis, variable**

In this section, competitive binding between CL16 and its molecular probe CL16-alkyne is examined by LC-MS/MS to characterize the target profile of CL16 in HCT116 cells. Despite the detection of CL16 engagement on RhoA Cys16 in previous MS experiment, CL16-alkyne serves as an enrichment probe to more precisely capture and profile the CL16-binding proteins in this section. Using a molecular probe enables substantiation of our findings and evaluation of the global selectivity of CL16 in CRC cells.

***Note:*** Unlike previous session, the protein samples from treated cells undergo CuAAC reaction for functionalization on Day 1 (Step 28–32). On Day 2 (Step 32–40), streptavidin agarose beads are added to enrich the probe-labelled proteins through overnight incubation. On Day 3 (Step 41–51), proteins are digested after on-beads capping. Lastly on Day 4 (Step 52–55), on-bead peptides are eluted for LC-MS/MS experiment, followed by analysis in Step 56–57. Figure 3C provides a schematic representation of the whole experimental setup. It is noteworthy that the development of molecular probes for other small molecules require prior knowledge of the structure-activity relationship (SAR) to append an alkyne handle at a suitable position for biorthogonal reaction. This approach based on inhibitor-probe competition for target identification can be applied to other small molecules.

#### Day 1

28. Incubate HCT116 cells with complete culture medium supplemented with DMSO or CL16 (25 μM) for 2 h at 37°C under 5% CO_2_.
29. Change to complete culture medium supplemented with DMSO or CL16-alkyne (50 μM) and incubate for 2 h at 37°C under 5% CO_2_.
30. Prepare cell lysates in PBS as described in step 14 (4 mg/mL; 2 mL per sample; *n* = 3).
31. Conjugate desthiobiotin onto CL16-alkyne modified proteins via CuAAC reaction.
  a. Add 240 μL of Click master mix solution to each sample.
  b. Incubate samples at 20°C–25°C for 1 h with gentle agitation.
32. Precipitate proteins with 12 mL of pre-chilled acetone at −20°C for 16–18 h.

***Note:*** This is an optional pause point. However, long-term storage of protein in acetone may cause denaturation. This protocol has not tested protein kept in acetone for longer than a weekend.

#### Day 2

33. Centrifuge samples at 13,200 × *g* for 10 min to precipitate proteins. Discard supernatant.
34. Resuspend protein pellet in cold methanol by probe sonication. Centrifuge samples at 6,500 × *g* for 5 min to precipitate proteins. Discard supernatant.
35. Repeat methanol washing once, i.e., step 34.
36. Resuspend protein pellet in 1.2% SDS denaturing solution by probe sonication. Boil the samples at 80°C for 5 min.

***Note:*** SDS crystallizes at low temperature. Avoid placing samples on ice starting from step 36.

37. Centrifuge the samples at 6,500 × *g* for 5 minutes. Discard any insoluble solids and save the supernatant which contains the labelled proteome.

**CRITICAL:** Finding a small blue pellet, due to Cu^2+^ salt, is normal. A large, white pellet suggests inadequate solubilization of proteins. Repeat sonication step from Step 36.

38. Prepare 100 μL streptavidin agarose slurry per sample in 5 mL PBS.
  a. Add 5 mL of PBS to a 15-mL Falcon tube for each sample.
  b. Transfer 150 μL streptavidin agarose slurry per sample to a spin column.
  c. Wash slurry with 1 mL PBS thrice.
  d. Resuspend agarose in 75 μL PBS (1:1 to bed volume).
  e. Transfer 100 μL washed slurry to each 15-mL Falcon tube.

***Note:*** Handle streptavidin agarose beads with cut-tip to avoid bead shearing.

39. Add the 1 mL supernatant solution from step 37 to the streptavidin agarose slurry in 15-mL Falcon tube.
40. Incubate the labelled proteome with streptavidin agarose slurry at 4°C for 16–18 h with head-to-head rotation.

#### Day 3

41. Re-solubilize any SDS crystal by shaking the samples at 20°C–25°C for 1 h.
42. Centrifuge the samples at 1,400 × *g* for 3 min. Discard supernatant.
43. Wash beads with 5 mL of 0.2% SDS by shaking the samples at 20°C–25°C for 10 min.
44. Centrifuge the samples at 1,400 × *g* for 3 min. Discard supernatant.
45. Transfer the washed beads using 250 μL of 6M urea to 1.5-mL Eppendorf tube with cut-tip.
46. Add 50 μL of TCEP solution to the mixture. Incubate at 65°C for 20 min with agitation.

***Note:*** If shaker is not available, gently tap the tubes to resuspend beads regularly. Avoid vortexing as this can shear the beads.

47. Allow the solution mixture to cool to 20°C–25°C. Add 50 μL of IA solution and incubate the mixture at 37°C for 30 min in the dark with agitation.
48. Dilute the reaction by adding 950 μL of PBS to each sample.
49. Centrifuge the samples at 1,400 × *g* for 3 min. Discard supernatant.
50. Resuspend beads in 200 μL of 2M urea.
51. Digest the proteome with 4 μL trypsin solution at 37°C for 16–18 h with constant agitation. Reconstitute trypsin as described in step 20.

#### Day 4

52. Transfer the bead suspension to a spin column. Collect digested peptides by centrifugation at 1,400 × *g* for 3 min.
53. Wash beads with 100 μL of PBS and collect the wash by centrifugation. Repeat once.
54. Dry and desalt the combined peptides.
55. Analyze peptides on LC-MS/MS as described in step 25.
56. Search the MS raw files using MSFragger (v3.7) with Uniprot human database (UP000005640).
  a. Specify digestion with trypsin with up to 3 missed cleavages.
  b. Include cysteine carbamidomethylation (+57.02146) as fixed modification, N-terminal acetylation (+42.01057) and methionine oxidation (+15.99491) as variable modifications.

***Note:*** Latest version of software or another search engine can be used.

57. Identify CL16-bound proteins.
  a. Remove possible false positives by filtering out proteins with low average intensity, small spectral count, or protein probability less than 1.
  b. Calculate the LFQ ratio by dividing the average LFQ intensity from DMSO-treated group with that from compound-treated group.
    ***Note:*** For proteins with zero LFQ intensity across triplicates in the compound-treated group and non-zero intensity across triplicates in the control group, assign a maximum capped value. For example, in our study of CL16, we assigned an LFQ ratio of 20 to these proteins.
  c. Evaluate the statistical significance for each protein by Student’s T-test based on LFQ intensities from triplicates per group.
  d. Visualize the result by volcano plot or other preferred forms. Example of CL16’s target profile is provided in Figure 3D.
    ***Note:*** The value of assigned LFQ ratio for proteins with all zero LFQ intensity within group depends on individual dataset.
    ***Note:*** You can cross-validate the identified protein targets with those found in the LC-MS/MS experiment described in steps 13–27 (e.g., Figure 3E).

### Pull-down and immunoblotting to assess RhoGTPase activity

**Timing: 3 days**

**Figure 4:**
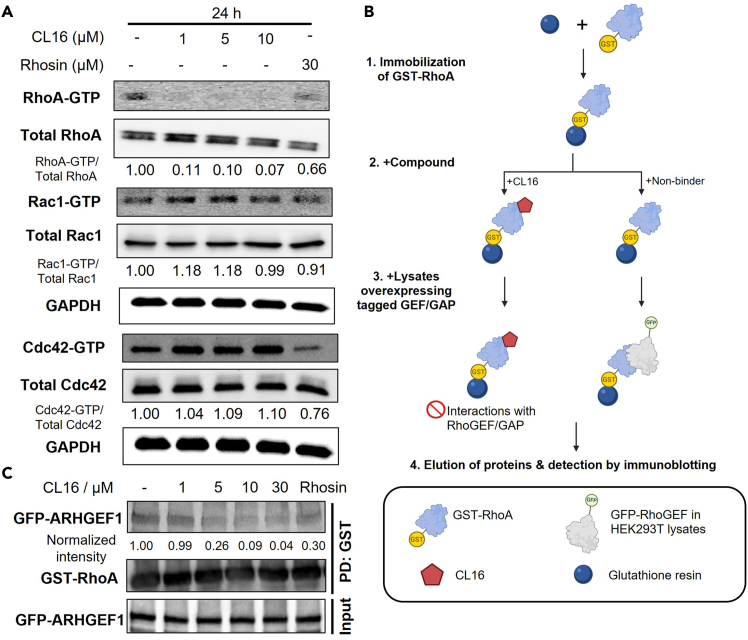
Biochemical assays to Investigate RhoA inhibition by CL16 (A) Expected results of RhoGTPase activity assay in which CL16 selectively impairs the activation of RhoA and not Rac1 and Cdc42.^1^ (B) Schematic diagram illustrating the workflow of the co-immunoprecipitation experiment. (C) Expected results of co-immunoprecipitation whereas CL16 interferes with interactions between RhoA and ARHGEF1.^1^

Having validated the RhoA engagement and selectivity of CL16, next we investigated activity of CL16 in CRC cells. This section describes the steps to detect active GTPase levels in cancer cells by pull-down using commercial kits followed by immunoblotting. The pull-down experiment confirmed CL16 as a selective RhoA inhibitor with minimal cross-reactivity in CRC cells (Figure 4A). The protocol described below is performed in HCT116 cells using CL16, but can be adapted for other adherent cell lines and compounds.

58. Seed HCT116 cells (2.5 × 10^6^ cells) into a 10-cm tissue culture dish in complete medium and allow them to grow for 16–18 h at 37°C under 5% CO_2_.
59. Aspirate medium. Incubate HCT116 cells with complete culture medium supplemented with DMSO, Rhosin (30 μM; positive control) or CL16 (0–30 μM) for 24 h at 37°C under 5% CO_2_.

***Note:*** Cells should reach *ca.* 90% confluency on the day of harvesting. Adjust cell number depending on treatment duration if necessary.

60. Prepare total cell lysates (0.5 mg/mL, 300 μL).
  a. Aspirate the culture medium. Add pre-chilled PBS to the cells.
  b. Aspirate the washing PBS.
  c. Harvest cell in 100 μL of ice-cold cell lysis buffer provided in the kit, supplemented with protease inhibitor cocktail.
  d. Clarify sample by centrifugation at 13,200 × *g* for 5 min. Save the supernatant.
  e. Quantify protein concentration (e.g., by Bradford, BCA or Lowry assay) and normalize protein sample with lysis buffer.

**CRITICAL:** Residual PBS in the washing steps 60b and 60c affects the pull-down efficiency.

**CRITICAL:** Do not use detergent-based cell lysis buffer as detergent denatures protein binding domain to beads.

61. Save 20 μL of total cell lysates as input.
62. Equilibrate Rho-RBD or PAK-PBD beads.
  a. Aliquot 10 μL of beads per sample in the same 0.6-mL Eppendorf tube.
  b. Wash beads with 500 μL of washing buffer provided in the kit.
  c. Centrifuge at 700 × *g* for 2 min to pellet beads.
  d. Resuspend beads in 5 μL of cell lysis buffer (1:1 to bed volume).

***Note:*** Handle all beads with cut-tip to avoid bead shearing.

63. Incubate a mixture of total cell lysates with 7.5 μL of beads at 4°C for 1 h with agitation.
64. Centrifuge at 700 × *g* for 2 min to pellet beads. Discard the supernatant.
65. Wash beads with 500 μL of washing buffer. Discard the supernatant.
66. Boil beads in 2× reducing sampling buffer at 90°C for 5 min.
67. Centrifuge at 5,000 × *g* for 5 min to pellet beads.
68. Collect supernatant containing eluted proteins for immunoblotting.

***Note:*** The activities of RhoA, RhoB and RhoC can be studied by Rhotekin-RBD beads due to their affinities for Rhotekin domain. The activities of Rac1 and Cdc42 can be studied by PAK-PBD beads. After pull-down, specific antibodies against each RhoGTPase are used to detect the enriched active protein by immunoblotting.

69. Quantify results by densitometric analysis for blots.
  a. Measure band intensity by ImageJ.
  b. Normalize the band intensity of the pull-down samples (the active RhoGTPase) by that of the input samples (the total RhoGTPase).

### Co-immunoprecipitation to study RhoA-GEF and RhoA-GAP interactions

**Timing: 2 days**

In addition to the activation of RhoGTPases, the physical interactions of RhoA with GEF or GAP proteins can be interfered by a RhoA inhibitor. This section outlines the steps to examine the *in* *vitro* interactions of RhoA with GEF/GAP by immobilizing recombinant GST-RhoA and co-immunoprecipitation (Figure 4B).

70. Transfer 15 μL of glutathione resin per sample to labelled tube and pre-equilibrated with 10× bed-volume of exchange buffer.
71. Prepare 10 μg of GST-RhoA in 200 μL of exchange buffer per sample.
72. Transfer the diluted GST-RhoA proteins into the labelled tube containing pre-equilibrated glutathione resin.

***Note:*** Do not vortex the beads to prevent sheading.

73. Incubate with DMSO, Rhosin (30 μM; positive control) or CL16 (1, 5, 10 or 30 μM) at 4°C for 1 h.
74. Dilute 120 μg of total cell lysates with overexpressed ARHGEF or ARHGAP in 200 μL of 1× exchange buffer per sample.

***Note:*** The preparation steps for cell lysates with overexpressed ARHGEF or ARHGAP are provided in the “before you begin” section.

75. Save 10 μL of the cell lysates as input.
76. Add the remaining lysate samples to the resin. Incubate at 4°C for 4 h with end-to-end rotation.
77. Pellet down the resin at 700 × *g* at 4°C for 2 min. Discard the supernatant.
78. Wash the resin with 1× exchange buffer thrice.
79. Boil the beads in 2× reducing sampling buffer at 90°C for 5 min to elute proteins.
80. Collect supernatant containing eluted proteins for immunoblotting.
81. Quantify results by densitometric analysis as described in step 69.

### Mouse CRC xenograft models to study *in vivo* antitumor activities of the compounds

**Timing: 1 month**

This section outlines the mouse experiment to investigate *in vivo* effect of CL16, with the focus on the construction of CRC xenografts and preparation of drug solution for administration.

82. Prepare CRC cell suspensions. Keep on ice during transfer to animal facility.
  a. Trypsinize CRC cells in culture dishes. Pellet cells by centrifugation at 200 × *g* for 5 min.
  b. Wash once with ice-cold PBS and pellet cells by centrifugation.
  c. Resuspend cell pellet in PBS.
  d. Count cells using a hemocytometer or automated counter.
  e. Inspect cell viability under a microscope using trypan blue staining. Ensure that > 90% of cells are viable.
  f. Dilute CRC cells with Matrigel/PBS to desired numbers. This protocol inoculates 2 × 10^6^ cells of HCT116, 3 × 10^6^ cells of HT29, or 1 × 10^6^ cells of SW620 per mouse.

***Note:*** This protocol uses 50% Matrigel/PBS, although PBS alone is also suitable for tumor implantation of these CRC cell lines. A higher percentage of Matrigel may improve cell viability but can make inoculation more difficult due to the increased viscosity of the suspension. Cell numbers and formulation should be optimized for other cancer models.

83. Implant tumor by subcutaneous injection at the dorsal side of BALB/c nude mice.
84. Monitor the mice closely until tumors reach 100 mm^3^ in size.
85. Freshly prepare CL16 in 1:1 PET/PBS solution for administration.
  a. Dissolve completely CL16 in PET solution by bath sonication.
  b. Dilute the solution with 1× volume of PBS.
  c. Mix by gentling up-and-down inversion of the vial/tube. Do not sonicate.

***Note:*** Prepare drug solution enough for *n* + 3 mice.

**CRITICAL:** Vortexing after mixing with PBS results in air bubbles which can affect subsequent injection. Allow the mixture to sit until air bubbles disappear. However, prolonged sitting may result in precipitation of compounds with low solubility.

86. Inject 200 μL of CL16 into PET/PBS solution intraperitoneally per mouse every two days until reaching humane endpoint. Measure the tumor volume and body weight of the mice throughout the experiment. Monitor health of the mice regularly.

**CRITICAL:** Withdraw the solution into syringe and inject it into mice slowly to avoid formation of air bubbles. PET/PBS solution is viscous.

87. Sacrifice mice and harvest tumors or organ tissues in compliance with regulations.
88. Analyze compounds effects by comparing tumor volume and weight, histology analysis, or other downstream assays (e.g., immunoblotting, single cell sequencing and etc).

## Expected outcomes

It is expected that this protocol will identify hit compounds selective for RhoA and successfully investigate their *in vitro* and in *vivo* biological effects. With minor adaptations, the gel-based ABPP and MS validation can be widely applied to other druggable targets whereas the biochemical assays for RhoA study may provide insight on other small GTPases.

Gel-based ABPP coupled with screening of a cysteine-reactive compound library allows identification of hit compounds targeting RhoA Cys16. A dose-dependent response of the compound binding to RhoA is critical for confirming that the compound is not a false positive in the 1^st^ screening experiment. The 2^nd^ screening focuses on identifying compounds that selectively target RhoA, rather than Rac1 or Cdc42, with Cys16 as the likely binding site because it is the only unique cysteine residue on RhoA. A representative result of ABPP experiments is provided in Figure 2B.

Regarding target engagement with RhoA protein, this protocol describes 2 different LC-MS/MS experiments: the unbiased profiling of CL16 modification of proteins in HCT116 cells and probe-enriched detection of CL16 protein targets. The 1^st^ experiment enables direct detection of CL16 modification on RhoA Cys16 in CRC cells in, but it is difficult to assess the target profile of CL16 as this experiment preferentially identifies modifications on abundant proteins in the CRC cells. Therefore, the 2^nd^ MS experiment using a molecular probe to enrich the protein targets of CL16 for detection should provide a more comprehensive detection of the protein targets of CL16 in CRC cells. In these experiments, labelling HCT116 cells with 25 μM of CL16-alkyne for 2 h and using 8 mg of protein input per samples yields about 50,000 peptides from 6,000 proteins. Filtering proteins to retain high-confidence entries allows targeting profiling over the remaining 2,800 proteins. Yet, it is noteworthy that direct detection of the binding site using molecular probe of CL16 is challenging due to its lower reactivity compared to promiscuous cysteine-reactive probes. As a result, we utilize both unbiased profiling of CL16 modification and CL16-molecular probe experiment to facilitate target identification with high confidence. Representative results of the two MS experiments are provided in Figures 3B and 3D.

In subsequent biochemical and biological assays, treatment with hit compounds from the screening experiment is expected to reduce active RhoA levels and disrupt its interactions with GEFs or GAPs (Figure 4), indicating a significant inhibition of RhoA activity. This underlies the potent anticancer effects of the compounds in cell-based experiments and mouse CRC models.

While this protocol reports the methods of identifying covalent inhibitors targeting RhoA Cys16, with minor adaptations, it can be applied to other druggable cysteines on other cancer-related proteins.

## Limitations

Although this protocol provides detailed procedures for identifying covalent inhibitors targeting RhoA Cys16, it is subjected to some limitations. Particularly, the screening is based on two-round gel-based ABPP experiments. These experiments are more cost-effective and accessible to most of the laboratories than the multiplex MS experiments,^14^ but they only provide information on *in vitro* binding between the compound and purified human RhoA protein. We anticipate that not all the hit compounds found in the screening experiment will be active in cell-based experiments or *in vivo*. Therefore, LC-MS/MS-based target engagement experiments are critical to confirm the binding of the hit compounds to RhoA in cancer cells. With our screening method which can eliminate non-binders from a large compound library, this should facilitate the development of specific covalent inhibitors.

In addition, the success of the screening experiment depends on the quality and the size of the cysteine-reactive compound library. While there are a number of commercially available sources, a random compound library should contain compounds that occupy different physiochemical spaces. These compounds should also comply with the Lipsinki’s Rule of Five to ensure they are “drug-like”. On the other hand, computational methods and artificial intelligence (AI) can be applied to predict optimal chemical structures for the protein of interest, enabling the establishment of a focused compound library. This approach can significantly increase the chance of developing potent and specific compounds for the protein target.

## Troubleshooting

### Problem 1

Obscure results from gel-based ABPP due to low/inconsistent signal from in-gel fluorescence, or no decrease in fluorescence intensity even when a reactive compound is tested (Step 5).

### Potential solution

• Prepare a single tube of diluted IA-Rh solution, before dispensing them into each sample to avoid discrepancy or technical errors in pipetting small quantity of solution during dilution.
• Optimize IA-Rh concentration to avoid insufficient labelling or saturation of signals. A pilot experiment can be performed using only the protein and IA-Rh at varying concentrations to determine the optimal IA-Rh concentration for the screening experiment.
• Constant and gentle agitation (Step 5) is important for the IA-Rh labeling in the small-volume reaction mixture. Avoid any solution mixture getting to the cap or wall of the tube to ensure consistency during the incubation of different samples.

### Problem 2

Obscure result from gel-based ABPP due to poor quality in silver staining gel or uneven protein loading (Steps 2 and 9).

### Potential solution

• Prepare a single tube of diluted protein solution, before dispensing them into each sample to avoid discrepancy or technical errors in pipetting small quantity of solution during dilution.
• Wash gel thoroughly with ethanol solution to get rid of excess fixative which can lead to huge background of gel during staining.
• Prepare new staining solution to avoid silver deposition.

### Problem 3

No CL16 modification on RhoA or low signals are detected in the LC-MS/MS-based proteomics experiments (Step 13 and 27b).

### Potential solution

• Increase protein input (Step 14).
• Freshly dissolve CL16 (or other hit compound) in dry DMSO before use. Water introduced during repeated freeze-thaw cycles may hydrolyze and inactivate the compound.
• Increase CL16 concentration (Step 13). Examine any toxicity of the compounds at this concentration and incubation time in advance.
• Fractionation of the tryptic digested peptides (e.g., by Pierce High pH Reversed-Phase Peptide Fractionation Kit from ThermoScientific) after desalting (Step 25). Then analyzes all the different fractions by LC-MS/MS.

### Problem 4

Low intensity or little peptides are detected in the MS experiments using the molecular probe (Steps 29, 30 and 43).

### Potential solution

• Increase protein input (Step 30).
• Increase concentration of CL16-alkyne (or other molecular probe; Step 29). Examine any toxicity of the probe at this concentration and incubation time in advance.
• Freshly dissolve CL16-alkyne (or other molecular probe) in dry DMSO before use. Water introduced during repeated freeze-thaw cycles may hydrolyze and inactivate probe.
• Ensure thorough washing of streptavidin beads after 16–18 h incubation with the protein samples (Steps 43 and 44) to wash out all the proteins with non-specific binding.
• Fractionation of the tryptic digested peptides (e.g., by Pierce High pH Reversed-Phase Peptide Fractionation Kit from ThermoScientific) after desalting (Step 54). Then analyzes all the different fractions by LC-MS/MS.

### Problem 5

Little enrichment of GTP-bound RhoA or other small GTPases in the pull-down assay, potentially due to low basal level of active proteins (Step 60).

### Potential solution

• Increase protein input.
• Optimize the amount of Rhotekin- or PAK-RBD beads.
• Include RhoA activator in the experiments as a positive control to confirm no technical errors in the experiments.

### Problem 6

Poor solvation of compounds in PET solution, or precipitation upon addition of PBS (Step 85).

### Potential solution

• Sonicate the compound with a longer time (Step 85).
• Examine the solubility of compounds in other biocompatible formulations. Of note, limit ethanol content to avoid dehydration, and limit DMSO (if used) to minimize toxicity. Monitor the health of mice injected with the solvent vehicle in a pilot experiment before the experiment with compound treatment.

## Resource availability

### Lead contact

Further information and requests for resources and reagents should be directed to and will be fulfilled by the lead contact, Dr. Clive Yik-Sham Chung (cyschung@hku.hk).

### Technical contact

Technical questions on executing this protocol should be directed to and will be answered by the technical contact, Dr. Tin-Yan Koo (u3548650@connect.hku.hk).

### Materials availability

CL16 and CL16-alkyne described in this study are available from the lead contact with a completed materials transfer agreement.

### Data and code availability

Raw proteomic data are available at the ProteomeXchange Consortium (identifiers: PXD053036 and PXD053041). Processed proteomic data, other examples, and source data for gel-based ABPP or RhoA-related biochemical assays are provided in the study by Koo et al.^1^

## Acknowledgments

C.Y.-S.C. acknowledges support from the Early Career Scheme (27315922) from University Grants Committee, and the Seed Fund for Basic Research for New Staff (202009185025) and Enhanced New Staff Start-up Research Grant from the 10.13039/501100003802University Research Committee and Li Ka Shing Faculty of Medicine, 10.13039/501100003803The University of Hong Kong. This work was also supported by the Centre for Oncology and Immunology under the Health@InnoHK Initiative funded by the 10.13039/501100003452Innovation and Technology Commission, the Government of Hong Kong SAR, China. T.-Y.K. and C.S.-H.H. acknowledge the receipt of a University Postgraduate Fellowship and a Postgraduate Studentship administered by The University of Hong Kong, respectively. H.Y.-Y.Y. acknowledges the receipt of a Hong Kong Postgraduate Fellowship Scheme and an HKU Presidential PhD Scholar Programme administered by the University Grants Committee and The University of Hong Kong, respectively. We acknowledge the support from the Proteomics and Metabolomics Core, Centre for PanorOmic Sciences (CPOS), Li Ka Shing Faculty of Medicine, The 10.13039/501100003803University of Hong Kong, for the MS experiments. We also thank Ms. Bonnie Yan and Mr. Ivan Lai, from the Department of Chemistry and Department of Microbiology, The University of Hong Kong, for their help in NMR experiments.

## Author contributions

T.-Y.K., conceptualization, visualization, investigation, methodology, writing – original draft, and writing – review and editing; C.S.-H.H., experiment, methodology, and writing – review and editing; H.Y.-Y.Y., experiment, methodology, and writing – review and editing; C.Y.-S.C., conceptualization, writing – review and editing, funding acquisition, and supervision.

## Declaration of interests

A patent application from C.Y.-S.C. and T.-Y.K. has been filed for CL16 and CL16-alkyne, and the US patent application number is 63/579,246.
